# Composition of Intestinal Microbiota in Two Lines of Rainbow Trout (*Oncorhynchus Mykiss*) Divergently Selected for Muscle Fat Content

**DOI:** 10.2174/1874285801812010308

**Published:** 2018-08-31

**Authors:** Karine Ricaud, Mickael Rey, Elisabeth Plagnes-Juan, Laurence Larroquet, Maxime Even, Edwige Quillet, Sandrine Skiba-Cassy, Stéphane Panserat

**Affiliations:** 1 INRA, Univ Pau & Pays Adour, E2S UPPA, UMR 1419, Nutrition, Métabolisme, Aquaculture, Saint Pée sur Nivelle, F-64310, France; 2 UMR 1313 INRA, AgroParisTech, Université Paris-Saclay, GABI, 78350 Jouy-en-Josas, France

**Keywords:** Rainbow trout, Gut microbiota, Selection, Fish lines, Adiposity, Aquaculture

## Abstract

**Background:**

Recently, studies suggest that gut microbiota contributes to the development of obesity in mammals. In rainbow trout, little is known about the role of intestinal microbiota in host physiology.

**Objective:**

The aim of this study was to investigate the link between intestinal microbiota and adiposity, by high-throughput 16S RNA gene based illumina Miseq sequencing in two rainbow trout lines divergently selected for muscle lipid content. Fish from these two lines of rainbow trout are known to have a differing lipid metabolism.

**Methods:**

Samples from the two lines (L for lean and F for fat) were collected from Midgut (M) and Hindgut (H) in juvenile fish (18 months) to compare intestinal microbiota diversity.

**Results:**

Whatever the lines and intestinal localisation, *Proteobacteria*, *Firmicutes* and *Actinobacteria* are the dominant phyla in the bacterial community of rainbow trout (at least 97%). The results indicate that richness and diversity indexes as well as bacterial composition are comparable between all groups even though 6 specific OTUs were identified in the intestinal microbiota of fish from the fat line and 2 OTUs were specific to the microbiota of fish from the lean line. Our work contributes to a better understanding in microbial diversity in intestinal microbiota of rainbow trout.

**Conclusion:**

Altogether, our study indicates that no major modification of the intestinal microbiota is induced by selection for muscle lipid content and associated metabolic changes. Finally, we identified members of core microbiota in rainbow trout.

## 1 INTRODUCTION

Intestinal microbiota plays a major role in animal’s health and host physiology, for example, in immunologic development or nutrient utilization. Microbiota can affect gut morphology and is known to stimulate the immune response and to protect against pathogens [1, 2]. The host can control the community by creating a niche for beneficial bacteria. In the other hand, bacteria provide to the host nutrients, extracellular enzymes, vitamins and fatty acids, not available without the bacterial community [3].

In fish, it is now well known that healthy gut microbiota is essential to promote host health and well-being and can reduce the proliferation of pathogenic bacteria [4, 5]. Culture-dependent methods allow to identify that the fish intestine harbors 10^7^ to 10^11^ bacteria/gram of intestinal content [6].

Furthermore, because of only a small part of intestinal bacteria is cultivable (about 1% regarding litterature) the use of molecular methods was developed to study microbial communities [7]. These molecular methods determinated that host species [8-10], lifecycle stage [11-13] and diet [14-16] play an important role in the composition of microbiota but interestingly in fish, due to the constant contact with water and sediments, microorganisms from water and soil may possibly have a even more important influence on bacterial composition [17]. In rainbow trout, intestinal bacteria are detectable before first feeding stage, 1 day post hatching, probably due to microorganisms coming from the aquatic environment [16]. Significant shift in the composition of gut microbiota are detected after first feeding. The dominant phyla regarding the diet are now well-known. Furthermore, the most abundant in fish fed plant based diet is the phylum *Firminutes* whereas *Proteobacteria* is dominant when fish are fed marine-based diet [16].

Two rainbow trout lines were divergently selected at INRA for low* vs *high muscle lipid content [17]. After 7 generations of selection, Fat line fish showed higher molecular capacities in hepatic gluconeogenesis (phosphoenolpyruvate carboxykinase gene, *pepck*), lipogenesis (glucose-6-phosphate dehydrogenase gene *i.e.* the main NADPH producer, *g6pd*), fatty acid bioconversion (D9-desaturase gene, *d9d*), and lower lipid β-oxydation (carnitine palmitoyltransferase gene, *ctp1*), compared to lean line fish [18-21]. Interestingly, it has been shown that the composition of gut microbiota differs in mammals (including human) microbiota depending on fat depots [22-24]. Indeed, the ratio *Firmicutes/Bacteroidetes* is closely related to fat deposits, increasing in obeses humans and decreasing with weight loss [23]. In ducks, studies comparing two genetic types with significant difference in capacities for fat storage allow to identify differences in the gut microbiota composition as reflected by the variable ratio *Firmicutes/Bacteroidetes* [25, 26].

We thus hypothesized that fatness and leanness in trout could be linked to differences in the composition of gut microbiota. In the present study, the gut microbiota was thus examined in the fat and lean INRA lines of rainbow trout using Illumina MiSeq high-troughput sequencing of the partial 16S rRNA gene in both midgut and hindgut segments of Gastrointestinal (GI) tract. To our knowledge, this is the first study to detect core and divergent microbiota associated to different muscle fat contents in rainbow trout lines.

## 2 MATERIALS AND METHODS

### 2.1 Ethics Statement

All experiment procedures involving fish were in accordance with EU legal frameworks relating to the protection of animals used for scientific purposes (Directive 2010/63/EU) and guidelines of the French legislation governing the ethical treatment of animals (Decree no. 2001-464, May 29^th^ 2001). It was approved by the ethics committee of INRA (INRA 2002-36, April 14, 2002). The INRA experimental station is certified for animal services under the permit number A64.495.1 by the French veterinary services, which is the competent authority.

### 2.2 Experimental Fish and Sampling Procedures

The study was performed using the two INRA lines of rainbow trout, Lean (L) line and Fat (F) line after 7 generations of two-way selection for low* vs *high muscle fat content [27, 28]. The two lines (18 months old and around 85g as an average) were maintained in tanks kept in a thermo(natural)-regulated at a constant water temperature of 7°C (INRA experimental fish farm, Lees Athas). At the time of the experiment, fish were fed at satiation with commercial diet T-2P Omega (Skretting, France). This commercial diet contains fish meal, fish oil from marine source and colza oil and soybean meal (analytical composition: crude proteins 42%, glucids 24% crude lipids, 20%, crude cellulose 3%, crude ash 6.5%).

Fish from different tanks were anaesthetized with an overdose of Benzocaine (0,031g/L). Liver and a sample of white muscle were dissected and immediately frozen in liquid nitrogen and stored at -80°C in order to control the status of the two fish lines. The digestive samples (midgut and hindgut contents) were collected by gently squeezing the intestinal organ 12h after the last meal. Midgut (M) and Hindgut (H) contents were immediately frozen and stored at -80°C before DNA extractions. This allowed to analyze 4 experimental groups: FM for fat line trout - midgut contents, FH for fat line trout - hindgut contents, LM for lean line trout - midgut contents, LH for lean line trout - hindgut contents with n=10.

### 2.3 Hepatic RNA Extraction and qRT-PCR

RNA extraction, from liver, was performed using the reagent Trizol^®^ (100 mg sample / 1 mL Trizol). The concentration was determined through the spectrophotometer NanoDrop 2000 and quality was determined by 1% agarose gel electrophoresis. Gene expression levels were determined by real-time RT-PCR (n=10; RNA samples per group). *pepck*, *cpt1*, *g6pd* and *pepck* mRNA levels were determined (previously described as differentially expressed between the two lines) using specific primers [18, 19, 29, 30]. An amount of 1µg RNA was reverse transcribed to cDNA with SuperScript III RNAseH-Reverse Transcriptase Kit (Invitrogen) with random primers (Promega). Real-time PCR was performed in the LightCycler 480 (ROCHE, Hercules, CA, USA). Quantitative PCR (Q-PCR) analyses for gene expressions were performed using a reaction mix of 6 μL per sample containing 2 μL of the RT produce (diluted cDNA), 0.24 μL of each primer (10 μmol/L), 3 μL Light Cycler 480 SYBR^®^ Green and 0.54 μL DNase/RNase-free water (5 Prime GmbH, Hamburg, Germany). Melting curves were systematically monitored (temperature gradient at 0.5°C/10 s from 55 to 94°C) at the end of the last amplification cycle to confirm the specificity of the amplification reaction. Each q-PCR run included duplicates of samples (reverse transcription) and negative controls (wells without reverse transcriptase, mRNA and cDNA). Relative quantification of target gene expression was performed using the ΔCT method. The reference gene elongation factor 1a (*ef1*α) was used for the normalization.

### 2.4 Muscle Lipid Analysis

Total lipids of the muscle and liver samples were extracted according to Folch * et al*. (1957), using dichloromethane instead of chloroform as the solvent and quantified gravimetrically.

### 2.5 Intestinal DNA Extraction

Total DNA from 20 fish (N= 10 per line) mindgut and hindgut samples was extracted combining mechanical, chemical and thermic lysis with an Ultra Turrax Digital Homogenizer IKA T-25 (Fisher Scientific, Illkirch, FR) and the QIAamp Fast DNA Stool Mini Kit (Qiagen Gmbh, Hilden, DE) according to the instructions of the manufacturer. The DNA sample was eluted with 50 µl of AE buffer (Qiagen Gmbh, Hilden, DE) and stored at – 20°C. The quantities and qualities of DNA extracted were measured with NanoVue Plus Spectrophotometer (GE Healthcare, Vélizy-Villacoublay, FR).

### 2.6 Barcoded PCR and Miseq Pyrosequencing

The PCR for sequencing were realized on the 16S rRNA gene according to the method described by Lluch ** et al**. [31] using MiSeq kit reagents v2 (2x250 bp pair ended reads). Amplicons from the V3-V4 regions of 16SrRNA genes were generated using specific bacterial primers 5’CTTTCCCT ACACGACGC TCTTCCGATCTA CGGRAGGCAGCAG 3’ and 5’GGAGTTCAGA CGTGTGCTCTTCCGA TCTTACCAGGGT ATCTAATCCT 3’. The preparation of amplicons was performed in a total volume of 50µL containing 1 U TAQ Polymerase and adequate 10 X PCR buffer (MTP Taq DNA Polymerase, Sigma), 200µM of dNTP (Sigma), 0.2µM of each primer and 2µL of DNA template. The amplification program consisted of an initial denaturation step at 94°C for 1 min and 32 cycles of denaturation at 94°C for 1 min, annealing at 63°C for 1 min and elongation at 72°C for 1 min. At the end, a final extension step at 72°C for 10 min was carried out. The quality of PCR products was controlled by electrophoresis. 2µL of PCR product were loaded on agarose gel (1% / TBE) with load Buffer for 30- 40 min at 135 V. Amplicons were then sent to the INRA genomic platform in Toulouse for sequencing. The amplicons were purified briefly using the magnetic beads Agencourt AMPure XP- PCR Purification (Beckman Coulter, Brea, CA, USA) following the 96-well format procedure, modified as follows: beads / PCR reactional volume ratio of 0.8 x and final elution volume of 32 μl using Elution Buffer EB (Qiagen). The concentration of the purified amplicons was controlled using Nanodrop 8000 spectrophotometry (Thermo Scientific). Single multiplexing was performed using a homemade 6-bp index, added to reverse primer during a second PCR with 12 cycles using forward primer (5’AATGATACGGCGACCACCGAGATCTACACTCTTTCCCTACACGAC 3’) and reverse primer (5’CAAGCAGAAGACGGCATACGAGAT-index-GTGACTGGAGTTCAGACGTGT 3’). The resulting PCR products were purified and loaded onto the Illumina MiSeq cartridge according to the manufacturer’s instructions. The quality of the run was checked internally using PhiX Illumina, and then each pair-end sequence was assigned to its sample with the help of the previously integrated index.

### 2.7 Sequence Analysis and Taxonomical Classification

A total of 260 580 16S rDNA sequences were sorted based on their respective barcodes representing the 39 collected hindgut and midgut samples. Sequences were filtered to remove sequences that (i) did not match the proximal PCR primer sequences (with 2 mismatches allowed), (ii) with a too short or too long sequencing length (less than 380 nucleotides or more than 500) and (iii) with at least one ambiguous base using FROGS developed by the French national Institute of Agriculture Research (INRA Toulouse, France [32]). Chimeric DNA sequences were detected using FROGS and removed. After trimming barcodes and adaptor sequences the average read length was 470 ± 25 nucleotides. A total of 234 534 reads were retained corresponding to 6015 sequences per sample. Reads were clustered into Operational Taxonomic Unit (OTU, cutoff of 0.05 using a furthest neighbor clustering) using SWARM [33] with the parameter d = 3. OTU taxonomic assignment was performed using the SILVA SSU Ref NR 119 database using the BLAT algorithm and RDP Classifier in FROGS software [32].

### 2.8 Statistical Analysis

The bacterial taxonomic classification data were normalized and standardized. The FROGS package, and VEGAN module of R (Community Ecology Package [34];) were used to generate relative abundance of intestinal microbiota and the diversity indexes from clusters (Chao1, Shannon, Simpson, inverse Simpson). The R software (R 3.1.3, R Development Core Team, 2015) was used to analyze all data in this study with a two-way ANOVA model, with, either hindgut or midgut as digestive content (A) and the line effect (TG) as fixed factors and the interaction between digestive and line effects (A x TG). Differences were declared significant at P ≤ 0.05. Data were reported as mean values with standard error.

## 3 RESULTS

We firstly characterized the two trout lines by analyzing previously known differences at biochemical (lipid muscle) and molecular (mRNA levels in liver of intermediary metabolism) (Table **1**) levels. As expected, F line fish had higher muscle lipid content than the L line fish (P=7.10^3^) with almost twice amount of total lipids. Moreover, *cpt1* mRNA levels were lower in the F line than in the L line whereas *pepck*, *d6d* and *g6pd* were expressed at higher levels in the F line than in the L line, confirming the metabolic differences between the two trout lines.

### 3.1 Microbial Community and Core Microbiota for all Samples (Irrespective of the Lines and Gut Segments)

**Fig. (1):**
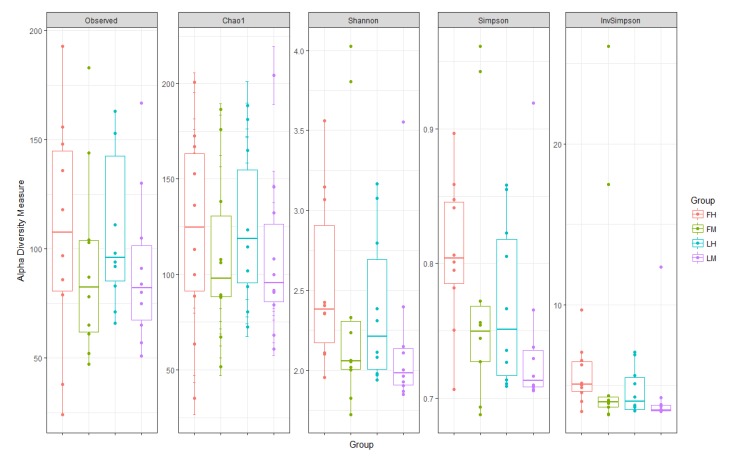
The Shannon, Simpson, Chao1, InvSimpson and observed indexes of fish communities regarding experimental group (M for midgut, H for hindgut, F for fat line and L for Lean line). The interquartile range is represented by the outer bounds of the boxes, the median is represented by the midline and the outliers are represented by the circles (O). The whiskers represent the minimum and maximum values.

The mean Chao1, the Shannon index, the Simpson and the inverse simpson were 117 ± 7, 2.4 ±0.1, 0.8 ± 0 and 5.6±0.7 respectively (Fig. **1**). The intestinal microbiota of the fish used in this study is constituted of a total of 16 phyla and is dominated by *Proteobacteria* (51.7% ± 1.7), *Actinobacteria* (30.5± 1.1) and *Firmicutes* (16.1± 2.6) accounting for 97% of all sequences. Phyla such as *Bacteroidetes*, *Tenericutes*, *Gemmatomonadetes, Fusobacteria*, *Spirochaeta, Cyanobacteria* represented less than 3%. To evaluate the microbiota composition at finer taxonomic levels, class distributions were analyzed. *Proteobacteria* were dominated by β- *Proteobacteria* (36.7% ± 1.3) α-*Proteobacteria* (7.7% ±0.3) and γ*-Proteobacteria* (4.9%±0.5). The class *Actinobacteria* accounted for all sequences of the *Actinobacteria* phylum (30.5% ± 1.1). *Clostridia* (10.9% ± 2.6) and *Bacilli* (4.8% ± 0.5) were the dominant classes in *Firmicutes*. Finally, 301 species were detected.

**Fig. (2):**
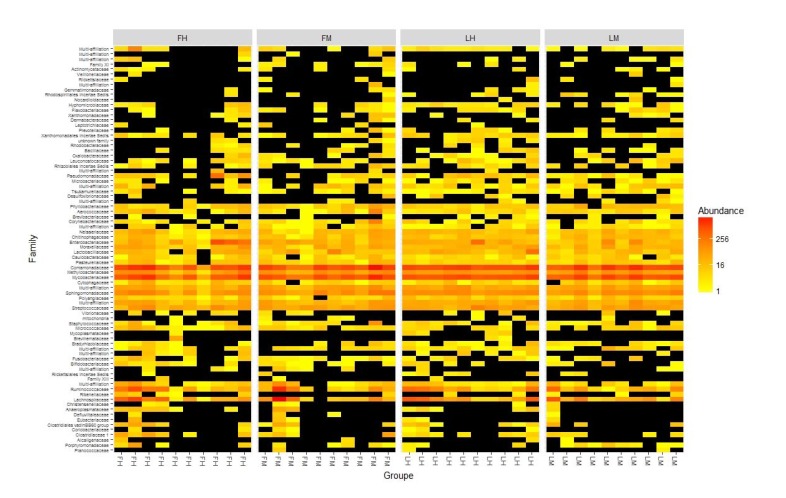
Heatmap of the bacterial community at the family levels regarding the segment (midgut and hindgut) and the genetic type (F or L). Samples and taxa were clustered according to Ward algorithm based on a Manhattan distance matrix.

225 OTUs were present in all experimental groups when 14 OTUs were present in all samples and composed the core microbiota. These 14 taxa were 8 *Proteobacteria*, 3 *Firmicutes*, and 2 *Actinobacteria*, representing 14 phylotypes at genus level: *Variovorax, Shingomonas, Methylobacterium, Alkanindiges, Neisseria, Mezorhizobium, Sorangium and Escherichia-Shigella for Proteobacteria; Lactococcus, Streptococcus and Lactobacillus for Firmicutes, Sediminibacterium for Bacteroidetes* and finally, *Mycobacterium and Rhodococcus for Actinobacteria.* Furthermore, Heatmap also allowed us to identify the two more abundant families: *Comamonaceae (Variovorax as genus level)* and *Mycobacteriaceae (Mycobacterium genus)* previously described in the core microbiota above (Fig. **2**). However, *Firmicutes* members of *Lachnospiracae* family, even if they were not present in all samples, are also important members.

### 3.2 Microbial Community Comparison Between F and L Lines and Between Midgut and Hindgut

The Chao, the Shannon, the Simpson diversity indexes were calculated and averaged by experimental group (Fig. **1**). Whatever the intestine localization (midgut or hindgut) or the line (F or L) or interaction between both conditions, there was no significant difference in diversity and richness indexes.

At phyla level, *Proteobacteria* was the dominant phyla in all experimental groups (FM:49.1%±4,8; FH: 52.4%±3,6; LM: 52.9%±3,0 and LH: 52.9%±3,0, respectively). As previously described for all samples, *Actinobacteria* (respectively 30.3%±2.9; 27.4%±2.1; 33.6%±1.7; 33.6%±1.7 for FM, FH, LM, LH) and *Firmicutes* were the other dominant phyla (FM: 19.2%±7.5 ; FH: 17.6%±4.1 ; LM: 12.1%±4.8 and LH: 15.3%±3.6, respectively).

**Fig. (3):**
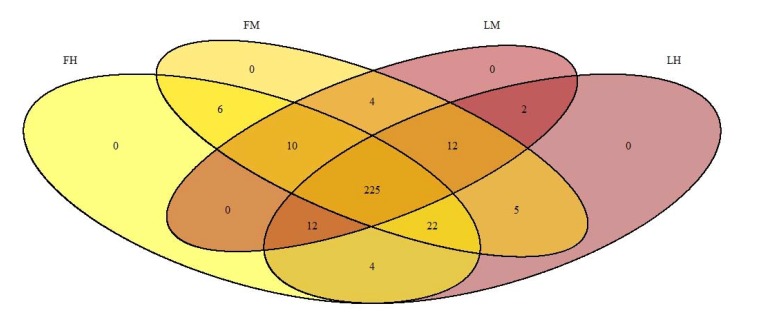
Venn diagrams showing compartmental core microbiota taxa distributions: 225 taxa were identified in all experimental groups.

**Fig. (4):**
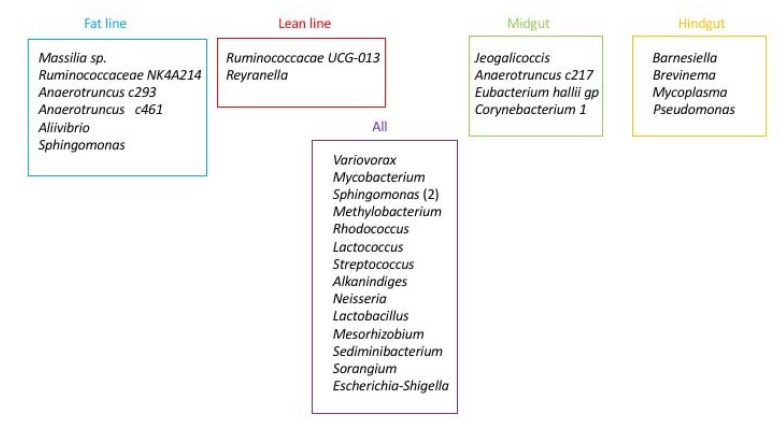
Specific microbiota in fat line, lean line, midgut and hindgut compartments at genus level and core microbiota of rainbow trout. gp means group for *Eubacterium halii* group.

Finally, the other phyla such as *Tenericutes*, *Bacteroidetes*, *Fusobacteria* and *Cyanobacteria*, represented less than 3% of the population in all experimental groups. At class level, as previously described for all samples, *Proteobacteria* are dominated by β- *Proteobacteria* in all experimental groups (respectively 35. 4%±3.6; 33.7%± 1.5; 39.8%± 2.3; 38.1%± 1.7 for FM, FH, LM, LH); then by α-*Proteobacteria* and γ*-Proteobacteria* (Table **2**). The class *Actinobacteria* accounted for all sequences of the *Actinobacteria* phylum groups (respectively 30.3%±2.9; 27.4%±2.1; 33.6%±1.7; 30.8%±1.4 for FM, FH, LM, LH). Regarding *Firmicutes* phyla, *Clostridia* was the major class in all experimental groups followed by *Bacilli* (Table **2**). Whatever the digestive content and the line or the interaction between both conditions, there was no statistical differences in the composition of gut microbiota, for neither diversity nor richness indexes (Table **2**, Fig. **1**). But Venn diagram allowed us to identify 6 OTUs specific to the fat line and only 2 OTUs specific to the lean line (irrespective of digestive contents) (Fig. **3**). In fat line, 3 taxa are from the phylum *Firmicutes* and family *Ruminococcae*, 2 were affiliated to *Anaerotruncus,* the last one not identified at genus level. The three other taxa were from *Proteobacteria* but from different families: *Oxalobacteraceae* (*Massilia* genus), *Vibrionaceae* (*Aliivibrio*) and *Sphingomonacae* (*Sphingomonas*). Furthermore, specific taxa associated to mindgut or hindgut content have also been identified: 4 for midgut contents and 4 for hindgut contents (Fig. **4**). Interestingly, 3 taxa from hindgut were identified to minority phyla: *Tenericutes*, *Spirochaetae* and *Bacteroidetes*. They have been affiliated respectively to families *Mycoplasmataceae* (*Mycoplasma* genus), *Brevinemataceae* (*Brevinema* genus) and *Prophyromonadacaee* (*Barnesiella*). The last one was a *Proteobacteria* from *Pseudomonacae* family and especially *Pseudomonas* genus. Furthermore, regarding specific midgut taxa, three were from *Firmicutes* phylum in three families: *Staphylococcae* (*Jeotgalicoccus*), *Ruminococcae* (*Anaerotruncus*), and *Lachnospiraceae* (*Eubacterium halii* group), and one *Actinobacteria*, in *Corynebacteriaceae* (*Corynebacterium* 1).

## 4 DISCUSSION

The microbiota of animals and human has been very well studied regarding its important role in host physiology [23, 35, 36]. Before the development of high-throughput sequencing method, the small part of cultivable bacteria in fish was a limit to study microbiota [37]. In this work, the microbiota of the gastro-intestinal (GI) tract was studied in two rainbow trout lines. To our knowledge, this is the first attempt using high—throughput sequencing to determinate the intestinal microbiota of rainbow trout differences in muscle fat content genetically determined. Our hypothesis was that a core microbiota would be identified in both lines, but that the diversity might be altered by the genetic characteristics which could be in relation with the different host metabolism, *i.e* glucose and lipid metabolisms, between the two lines (18–21). The idea of a core microbiota (bacterial taxa identified in all samples regardless the diet and environment) being responsive for the functionality of GI tract microbiota was purposed recently [38, 39]. If this concept has been very well documented in mammals, recent works in fish suggested the existence of a core microbiota in zebrafish [38], Atlantic cod [39], Atlantic Salmon [40] and rainbow trout [15]. A previous work in juvenile rainbow trout also identified a core microbiota, irrespective of the diet and rearing density, and composed mainly of *Bacilli, Alphaproteobacteria, Gammaproteobacteria* [15].

As previously suggested by Sullam * et al* [8], the gut microbiota of fish may not only be reflected by water microbiota. Furthermore, colonization of germ-free zebrafish with a *Firmicutes* dominant gut mouse microbiota triggers a zebrafish microbiota dominated by *Proteobacteria* (which is the dominant phylum in zebrafish) suggesting that the host selects its microbiota whatever the initial composition of inoculates [41]. Then, other studies comparing microbiota of fish reared in natural and artificial environment allowed to detect only few differences among environments [38, 40].

In the present study, we identified 225 OTUs present in all experimental groups (irrespective of the fish lines) and 15 OTUS in all samples suggesting a high variability between samples as previously described in microbiota studies but allowing us to identify a core microbiota. The most dominant phylum was *Proteobacteria*, followed by *Actinobacteria* and *Firmicutes* in accordance with previous works in rainbow trout and Atlantic salmon [42, 43]. It is clear now that gut microbiota in fish clearly differs from mammal where *Firmicutes* and *Bacteroidetes* are dominant [22-24]. It is also commonly accepted now that *Firmicutes* are dominant in plant based fed fish when *Proteobacteria* is the most abundant in marine fed fish [16]. The fish in our study were fed with a commercial diet containing fishmeal and fish oils as well as plant ingredients; we found an average of 51% of *Proteobacteria*., then *Actinobacteria* and *Firmicutes* are the two other major phyla reflecting the presence of both plants ingredients and fishmeal (or fish oil) in the diet. In humans, it has been shown that *Firmicutes* are specialized in the degradation of non-starch polysaccharides [44]. Gene pathways identified in members of *Spirochaetes* and *Firmicutes*, as explained above, are implicated in fermentation of non-starch carbohydrates* via *the anaerobic glycolytic pathway [45]. This result in the production of short chain fatty acids (SCFA) such as butyrate, proponiate and acetate. Interestingly, several fish species, in particular rainbow trout produced high concentrations of SCFA which can be partially explained by bacterial groups in microbiota [46]. Furthermore, the major OTU identified here in the core microbiota, *Variovorax* genus from *Proteobacteria* has already been identified in the core microbiota of *Salmo salar* [47]. Then, members of *Variovorax* were also recently identified in soil and associated to plant root and are known to be able to degrade lignocellulose [48, 49]. This is the same for *Sphingomonas* also member of the core microbiota in our study and in *Salmo Salar* [47]. Finally, the capacity of trout intestinal microbiota to use non-starch carbohydrates to provide energy (as SCFA) to the host metabolism should be studied in the future.

Other members of *Proteobacteria* were identified in the core microbiota of rainbow trout: *Methylobacterium* that was identified as associated to a QTL(quantitative trait loci) identified in link with protection against pathogens [50]; *Escherichia-Shigella*, already described in intestinal microbiota of salmon and rainbow trout [16, 40]; *Alkanindiges*, described in intestinal microbiota of Atlantic mackerel [51] and *Neisseria* which, was once isolated in farmed fish in Ghana [52]. To our knowledge, this study represents the first evidence of *Sorangium* and *Mezorhizobium* as members of core intestinal microbiota in fish. *Sorangium* has been detected in water of tank containing rainbow trout and is known to be able to produce geosmin [53]. *Mezorhizobium* has been also described in recirculating aquaculture system water [54]. Thus, our study suggests that water environment bacteria can colonize fish intestine successfully over time and not only during the first stage of gut colonization as previously suggested [12, 16, 55]. To clearly provide evidence to this hypothesis, studies on younger animals are required.

Members of *Firmicutes*, especially, members of *Clostridia* class and *Lachnospiracae* family have been identified as proteolytic bacteria and could ferment amino acids. Several authors suggested the involvement of the intestinal microbiome in protein metabolism, especially in crucian carp where intestinal microbiota is responsible for 45% of peptidase production [46, 56]. Here, the presence of potential proteolytic bacteria in intestinal microbiota (but not in core microbiota) should be investigated in further experiments to correlate microbiota diversity to digestive enzyme activities.

Then, we identified as core microbiota members of *Firmicutes,* different lactic bacteria: *Lactococcus* also detected in salmon during winter (with temperatures closed with the ones in our rearing location)*;* then *Streptococcus* and *Lactobacillus*, lactic bacteria already detected in intestinal microbiota in salmon and rainbow trout [16, 40].

Moreover, even if *Actinobacteria* phylum was already identified in rainbow trout microbiota, the important average proportion identified in our study (30,5%) is quite surprising. Almost all sequences are related to *Mycobacterium*, where several species can be pathogen in fish [57] and here, the sequence affiliated suggested a close relationship with *M. Frederiiksbergense*, a species already found in soil in Denmark [58]. As previously described, other bacteria already identified in soil have been detected in this study, suggesting that aquatic environment (water and soil) could be partially explained microbial diversity in fish. However, Asakura * et al* [59] identified *Mycobacterium spp.* as an important cluster to explain specific bacterial composition in omnivorous feeding fish. *Rhodococcus* another member of *Actinobacteria* phylum that has been identified as a member of core microbiota of salmon and detected in rainbow trout regardless the diet [16, 47] was also present in the core microbiota of our rainbow trout:

A member of *Bacteroidetes* and especially *Sediminibacterium* genus was also detected in our study as core microbiota member and identified in water tanks with tilapia larvae. Interestingly, *Sediminibacterium* was dominant in rainbow trout before the first feeding but the abundance decreased with feeding and age [16]. Finally, most of the bacterial groups identified were already identified in other studies in rainbow trout or salmon. Furthermore, new species identified as environment bacteria were also detected suggesting the importance of environmental or water bacteria.

In our study, our main objective was to compare intestinal microbiota between the two trout lines which differ by their lipid muscle content. Our hypothesis was that, as previously described in obese and lean mammals [22-24], microbiota diversity could be different between the lines and correlate with different metabolic phenotypes.

Unlike studies in mammals, no statistical differences were identified between the two trout lines regarding diversity richness indexes and bacterial composition. This could be partially explained with the age of animal, which can have a more stable microbiota than younger fish [26]. However, interestingly, we identified 6 OTUs only identified in samples from fat line and 2 OTUs from samples issued from lean line. These OTUs were already described in intestinal microbiota in several animals but never identified as specific in both line was already identified as biomarkers of obesity in mammals.

However, further investigations are necessary to definitively conclude to the absence of differences for the microbiota between the two lines. Indeed, the fish were reared at low temperature (7°C). At this temperature, the feed intake is lower compared to trout reared at 18°C [60] which may limit the effects of feeding on the microbiota composition and thus the possible differences of microbiota between the two lines. Further studies at higher temperatures are thus necessary to analyze the relation between diets and microbiota in the two lines. Regarding midgut and hindgut contents, few OTUs were identified as discriminant between the two segments. It was not possible to conclude because the sequences were minority and not described in bibliography.

## CONCLUSION

In conclusion, our study confirmed that the core microbiota between the fat and lean rainbow trout lines remained similar, independently to their genetic differences and the intestinal sections (midgut and hindgut) suggesting that in our experimental conditions diet and environment can play a more important role in microbial diversity than genetics. Three phyla, *Proteobacteria, Actinobacteria* and *Firmicutes* were dominant in all the fish samples. No statistical differences were detected in microbial diversity between the experimental groups but a few membership community members were identified specifically to genetic lines and digestives contents. Further investigations are necessary to clarify the potential role of these specific bacterial groups and to understand the functional microbiota in fish and improve gut health and metabolism in fish.

## ETHICS APPROVAL AND CONSENT TO PARTICIPATE

It was approved by the ethics committee of INRA (INRA 2002-36, April 14, 2002). The INRA experimental station is certified for animal services under the permit number A64.495.1 by the French veterinary services, which is the competent authority.

## HUMAN AND ANIMAL RIGHTS

Humans did not participate in this research. All experiment procedures involving fish were in accordance with EU legal frameworks relating to the protection of animals used for scientific purposes (Directive 2010/63/EU) and guidelines of the French legislation governing the ethical treatment of animals (Decree no. 2001-464, May 29th 2001).

## CONSENT FOR PUBLICATION

Not applicable.

## Figures and Tables

**Table 1:** Characterization of the fat and lean rainbow trout lines. Muscle lipid content and levels of mRNAs for genes previously known to be hepatic metabolic biomarkers for the two lines (Skiba-Cassy * et al*, 2009; Panserat * et al*, 2009, Kamalam * et al*, 2012; Kamalam * et al*, 2013) are shown.

	**Lean Lines**	**Fat Lines**	**P values**
Hepatic Gene expression * (AU) **(n=10 fish per group)**			
*cpt1*	1.24 ± 0.58	0.62 ± 0.49	0.02
*d6d*	0.85 ± 0.14	1.23 ± 0.39	0.01
*g6pd*	0.84 ± 0.34	1.16 ± 0.31	0.04
*pepck*	0.87 ± 0.26	1.28 ± 0.31	4 10^-3^
**Lipid muscle content (%)** (n=6 fish per group** –			
**Fish Weight (g)** **Liver Weight (g)**	4.33 ± 0.71 89,6±2,3 0,81±0,03	8.06 ± 1.21 86,8±2,6 0,87±0,04	7 10^-3^ N.S N.S

* EF1α: reference gene ** Folch measure *cpt1*: carnitine palmitoyltransferase type 1; *d6d*: Δ6-desaturase; *g6pd*: glucose-6-phosphate dehydrogenase; *pepck*: phosphoenolpyruvate carboxykinase.

**Table 2:** Percentage distribution of sequences (%) evaluated at the phylum and class levels to the total number of sequences in both midgut and hindgut independently to the genetic type (F or L).

In %	FM	FH	LM	LH	*P*
	(n=10)	(n=10)	(n=9)	(n=10)	
*Firmicutes*	19,2±7,5	17,6±4,1	12,1±4,8	15,3±3,6	N.S
*Bacilli*	5,6±1,2	5,0±1,1	3,7±0,3	4,7±0,7	N.S
*Clostridia*	13,1±7,6	12,2±4,6	8,1±4,9	10,4±3,7	N.S
*Proteobacteria*	49,1±4,8	52,4±3,6	52,9±3,0	52,5±2,4	N.S
*Alphaproteobacteria*	8,1±1,0	6,9±0,6	8,3±0,6	7,7±0,4	N.S
*Betaproteobacteria*	35,4±3,8	33,7±1,5	39,8±2,3	38,1±1,7	N.S
*Gammaproteobacteria*	3,2±0,5	9,7±3,9	2,5±0,5	4,4±1,5	N.S
*Deltaproteobacteria*	0,4±0,1	0,4±0,1	0,5±0,1	0,5±0,1	N.S
*Actinobacteria*	30,3±2,9	27,4±2,1	33,6±1,7	30,8±1,4	N.S
*Actinobacteria*	30,3±2,9	27,4±2,1	33,6±1,7	30,8±1,4	N.S
*Bacteroidetes*	0,9±0,2	1,1±0,1	1,2±0,1	0,9±0,1	N.S
*Tenericutes*	0,2±0,2	0,2±,1	0,0±0,0	0,2±0,0	N.S
*Spirochaetea*	0,0±0,0	0,8±0,8	0,0±0,0	0,0±0,0	N.S
*Fusobacteria*	0,1±0,0	0,1±0,1	0,1±0,0	0,1±0,0	N.S
*Cyanobacteria*	0.0±0.1	0.0±0	0.0±0	0,1±0,0	N. S
Others (<0.1%)	0.2	0.4	0.1	0.1	N.S
